# Cardiovascular Effects of Long-Term Treatment with Enhanced External Counterpulsation in Patients with Ischemic Heart Failure: Randomized, Placebo-Controlled, Open-Label Clinical Trial

**DOI:** 10.3390/jcdd12090352

**Published:** 2025-09-13

**Authors:** Alexey S. Lishuta, Olga A. Slepova, Nadezhda A. Nikolaeva, Yuri N. Belenkov

**Affiliations:** Department of Hospital Therapy No. 1, I. M. Sechenov First Moscow State Medical University (Sechenov University), 119991 Moscow, Russia; slepova_o_a@staff.sechenov.ru (O.A.S.); nikolaeva_n_a@staff.sechenov.ru (N.A.N.); belenkov_yu_n@staff.sechenov.ru (Y.N.B.)

**Keywords:** long-term treatment, enhanced external counterpulsation, ischemic heart failure, vascular effects

## Abstract

(1) Background. Although treatment with enhanced external counterpulsation (EECP) in patients with ischemic chronic heart failure (CHF) is pathophysiologically justified, its long-term vascular effects remain insufficiently defined. We aimed to study the vascular effects of long-term complex treatment (36 months) including EECP in patients with ischemic CHF, and to examine the relationship between these effects and clinical outcomes. (2) Methods. A total amount of 120 patients with ischemic CHF were randomized to receive one course of EECP per year (35 h; Group 1), two courses of EECP per year (70 h; Group 2), or one course of placebo-counterpulsation per year (35 h; Group 0;). For a period of 36 months, all patients underwent annual assessments including transthoracic echocardiography, nailfold videocapillaroscopy, finger photoplethysmography, applanation tonometry, exercise tolerance testing, and clinical outcome monitoring. (3) Results. Compared to the placebo group, long-term EECP treatment in patients with ischemic CHF, was accompanied by a significantly greater increase in exercise tolerance (∆23.5–45.0% vs. 7.0%; *p* < 0.001) and improvements in left ventricular ejection fraction (∆9.9–19.6% vs. 5.6%; *p* < 0.001) and myocardial stress (decrease in NT-proBNP level ∆−80.4–−82.4% vs. −75.8%; *p* < 0.001), as well as both functional and structural vascular parameters (*p* < 0.001). The effect size depended on the annual number of EECP courses. The highest event-free survival was found in Group 2. At 36 months, improvement of vascular parameters emerged as stronger predictors of reduced cardiovascular event risk compared to the 12-month. (4) Conclusions. Long-term EECP treatment of patients with ischemic CHF improves both functional and structural vascular parameters, with an increasing role of their improvement in reducing the risk of cardiovascular events after 36 months.

## 1. Introduction

Chronic heart failure (CHF) is a common problem, with an increased incidence due to a longer life expectancy in the current population [1]. Despite the periodic implementation of new approaches to therapy, CHF remains associated with a high rate of adverse outcomes and a significant deterioration in the quality of life of this category of patients [1].

CHF is a clinical syndrome that arises from an imbalance between vasoconstrictor and vasodilating neurohormonal systems as a result of an impaired ability of the heart to fill and/or empty, accompanied by insufficient perfusion of the organs and systems necessary for tissue metabolism. CHF manifests as complaints of shortness of breath, weakness, palpitations, increased fatigue, and, as the disease progresses, congestion [2,3]. Coronary artery disease (CAD) is the main cause in 2/3 of CHF cases with a low ejection fraction and in almost half of cases with a mildly reduced ejection fraction [4].

Vascular disorders play a critical role in the development and progression of CHF. The structural vascular changes that occur in both macro- and microvascular components of CHF include vascular remodeling (e.g., hypertrophy, fibrosis and changes in vascular smooth muscle function, a reduction in capillary density). Microvascular dysfunction is a determining factor in the development of ischemic heart failure, and coronary endothelial function plays a key role in the regulation of blood flow in both the epicardial and intramyocardial microcirculation [5]. Functional impairment of the vascular system in CHF is primarily associated with endothelial dysfunction and systemic inflammation, both of which contribute to changes in vasomotor reactions and impaired tissue perfusion [6].

In elderly patients with CH, myopathy is associated with decreased physical performance, which can be explained by a reduction in capillary density and impaired vasodilatory responses in skeletal muscles [7]. Diminished microcirculatory function further complicates the overall clinical course of CHF, exacerbating symptoms such as fatigue and exercise intolerance.

These factors underlie the search for additional effective treatment methods that could increase the overall effectiveness of treatments for patients with ischemic CHF when added to the standard conservative and invasive strategies.

Enhanced external counterpulsation (EECP) is used in the treatment of patients with CAD, including cases complicated by the development of CHF [8]. The advantages of EECP are its non-invasiveness and safety. The positive effect of EECP on left ventricle (LV) systolic function, exercise tolerance, and quality of life in patients with CAD, including those with systolic dysfunction, has been proven [9,10]. However, the long-term effects of EECP treatment on the morphofunctional state of the vessels, which are the main target of this method of treatment and an important component in the pathophysiology of CAD and CHF, have not been sufficiently investigated.

In this study, we are aimed to study the vascular effects of long-term complex treatment, including EECP, in patients with CHF of ischemic genesis and the relationship between these effects and clinical outcomes.

## 2. Materials and Methods

### 2.1. Study Design and Population

The open-label, randomized, placebo-controlled EXCEL (Long-term Effects of enhanced eXternal CountErpuLsation; NCT05913778) enrolled 120 patients aged 40 to 75 years with CAD complicated by CHF (with a reduced or mildly reduced LV ejection fraction [EF]), who did not meet the exclusion criteria and signed an informed consent form [11]. All patients had been receiving optimal medical therapy (OMT) for CHF and CAD for at least 3 months at the time of inclusion. Medical treatment was prescribed by the treating physician, following actual guidelines but not standardized. All patients had renin-angiotensin system inhibitors (57.8%—angiotensin-converting enzyme inhibitors, 6.7%—angiotensin II receptor blockers or 35.6%—angiotensin receptor neprilysin inhibitor), beta-blockers—100%, ivabradine—11.1%, digoxin—10.0% (patients with atrial fibrillation), mineralocorticoid receptor antagonists—97.8% (2 patients had contraindications), sodium-glucose cotransporter-2 inhibitors—42.4%, statins—100%, ezetimibe—77.8%. The need and doses of diuretics depended on the patients’ clinical status. Patients did not regularly participate in a rehabilitation program. The study protocol complied with the Declaration of Helsinki and was approved by the Ethics Committee of Sechenov University. The patients were split into 3 equal groups (1:1:1). In the first stage after randomization, the long-term (12 months) effects of EECP therapy were compared to placebo counterpulsation.

Patients in Group 1 (*n* = 40) received 1 course of EECP annually, consisting of 35 one-hour sessions, 5 times a week for 7 weeks; compression pressure 220–280 mmHg) in addition to OMT. Patients in Group 2 (*n* = 40), along with OMT, underwent 2 similar courses of EECP annually with an interval of 6 months between them. The control Group 0 (n = 40) received an annual course of placebo counterpulsation with a subtherapeutic level of compression, consisting of 35 one-hour sessions, 5 times a week for 7 weeks; compression pressure 80 mmHg) in addition to OMT.

**Figure 1:**
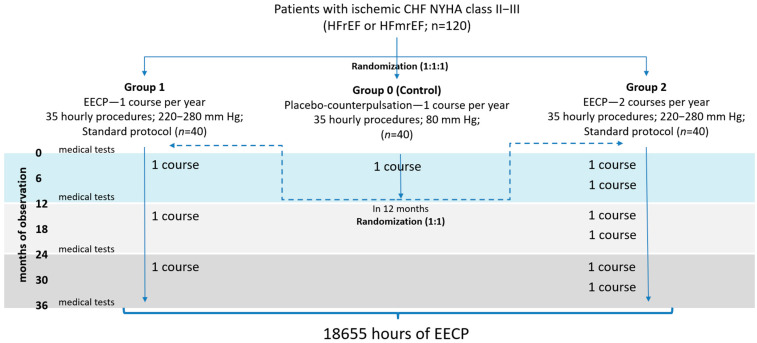
EXCEL (Long-term Effects of enhanced eXternal CountErpuLsation) study design. CHF, chronic heart failure; NYHA, New York Heart Association; HFrEF, heart failure with reduced ejection fraction; HFmrEF, heart failure with mildly reduced ejection fraction; EECP, enhanced external counterpulsation.

In the second stage of the study, the long-term effects of EECP therapy were assessed over a 36-month period. After 12 months, the patients in the control group were randomized in a 1:1 ratio to Group 1 or 2. Observations of the patients in these groups continued for 36 months (Figure 1).

The effects of EECP treatment on indicators of the structural and functional state of the vessels (Table 1) were studied in addition to an assessment of the dynamics of exercise tolerance, myocardial stress markers (NT-proBNP), and LV systolic function (echocardiography). All investigations were conducted at baseline and after 12, 24, and 36 months of observation (at the end of each year of observation–before the next EECP course).

Transthoracic echocardiography was performed using a Phillips IU22 (Philips Helthcare, Eindhoven, The Netherlands) device according to a standard protocol, with assessments of the volumetric dimensions of the LV (end systolic volume, end diastolic volume), thickness of the interventricular septum and posterior wall of the LV, LV myocardial mass index (MMI), and LV systolic (EF) and diastolic (E/A) function [12].

Nailfold videocapillaroscopy was performed using a Capillaroscan-1 device (LLC New Energy Technologies, Moscow, Russia). The resting capillary density (RCD) and its microcirculation functional reserves (capillary density in reactive hyperemia and venous occlusion tests) were estimated at a magnification of ×200, which reflects endothelial function and the possibilities of capillary adaptation [13].

Finger photoplethysmography was performed using an Angioscan-01 (Angioscan-Electronics, Moscow, Russia). The study protocol included an analysis of the shape of the volumetric pulse wave, its intervals and amplitudes, and the phases of heart activity and allowed for assessments of the structural and functional parameters of both large arteries and the microcirculation [14].

Applanation tonometry (A-pulse CASPro, Healthstats International Pte. Ltd., Singapore) is a non-invasive research method that allows for assessments of central aortic systolic pressure (CASP) and the radial augmentation index (rAI), which indicates the degree of arterial stiffness [15].

Exercise tolerance was measured using the 6 min walk test (6 MWT), which is a validated and clinically informative method of assessment of the physical activity of patients with CHF [16].

The Charlson Comorbidity Index, a validated tool that allows quantitative description of the level of comorbidity, was used to assess the combined impact of concomitant chronic diseases on the general condition of patients. The use of the index is especially relevant in long-term clinical observations, since it allows predicting the likelihood of remote adverse outcomes [17].

In addition, adverse events associated with the progression of CAD including revascularization, acute coronary syndromes, myocardial infarction, deaths from all causes–were registered, forming the vascular composite endpoint (VCE). Moreover, new-onset episodes of atrial fibrillation or type 2 diabetes mellitus, decreased renal function, and decompensation of CHF with hospitalization were assessed. These events together constituted the overall composite endpoint (OCE).

### 2.2. Statistical Analysis

Quantitative variables with non-normal distributions were described using medians (Me) and interquartile range (Q25–Q75); while those with normal distributions were presented as means and standard deviation (M ± SD). Categorical variables were described by their absolute (n) and relative (%) frequencies. Quantitative variables in 2 groups with non-normal distributions were compared using the Mann–Whitney U test. Comparisons of dependent observations were performed using the Wilcoxon test, which provides a reliable assessment of changes in dynamics. This was used in situations involving an analysis of 3 or more groups. Three or more groups with a non-normal distribution were quantitatively compared using the Kruskal–Wallis test. The event-free survival was estimated using the Kaplan–Meier curves, allowing the visual comparison between groups. A stepwise approach with backward elimination of predictors using the Wald test was applied to construct logistic regression models. During the analysis, only statistically significant factors that passed the predetermined significance level were included in the model. An ROC analysis was performed for quantitative variables that were considered significant during the analysis. The results were used to calculate the area under the curve (AUC) with a 95% confidence interval, and the corresponding curves were constructed to visualize the diagnostic value and threshold levels of the studied parameters. When relative variables were compared between groups, the odds ratio (OR) was used as a measure of effect, reflecting the probability of achieving the target outcome depending on the assigned therapeutic group. *p* values of <0.05 were considered statistically significant. The statistical analysis was performed using SPSS Statistics 26.0 (IBM Corp; Armonk, New York, NY, USA) and StatTech v.3.0.5 (StatTech LLC, Moscow, Russia).

## 3. Results

**Figure 2:**
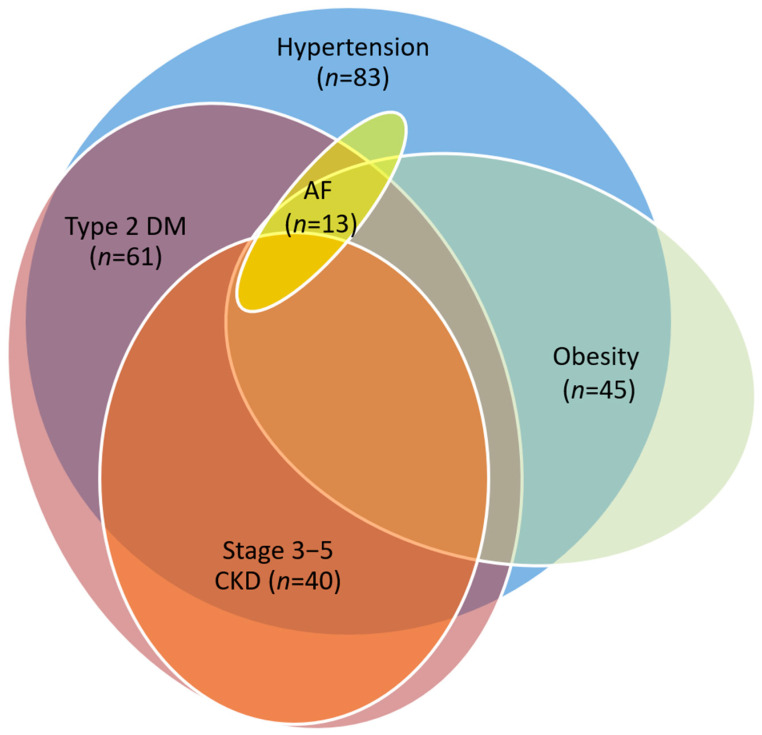
Comorbid conditions in all patient groups (n = 120). AF, atrial fibrillation; DM, diabetes mellitus; CKD, chronic kidney disease.

All patients with ischemic CHF had various comorbid conditions, including hypertension, type 2 diabetes, obesity, stage 3–5 chronic kidney disease, and atrial fibrillation (Figure 2). The basic clinical and demographic characteristics of the study groups are summarized in Table 2.

A significant improvement in exercise tolerance was identified during the observation period in all groups compared to the baseline (Table 3). In Groups 1 and 2, after just 3 months, the 6 MWT distance increased significantly more than in Group 0. The maximum increase in physical tolerance was found in Group 2 and was maintained throughout the study (36 months).

Assessments of the dynamics of the NT-proBNP level showed a significant decrease in all groups. The NT-proBNP levels in the EECP patient groups (1 and 2) were significantly lower than those in the placebo counterpulsation group (Table 4). Throughout the study period, the NT-proBNP level in Group 2 (70 h of EECP per year) was significantly lower than that in Group 1 (35 h of EECP per year). In addition, patients in Group 2 showed a significant increase in their glomerular filtration rate over the observation period (36 months), whereas Group 1 showed a decrease.

The improvement in exercise tolerance among the patients was accompanied by a significant increase in LV systolic function. At the same time, patients in Group 2 (70 h of EECP per year) showed a significantly greater increase in LV EF compared to the other groups (Table 5), and these advantages were maintained throughout the observation period (36 months). A significant decrease in the LV MMI compared to baseline values was found in each group, although the differences between the groups were not statistically significant during the study.

Significant dynamics for the studied parameters (stiffness index [SI], reflection index [RI], phase shift [PS], occlusion index [OI]) of finger photoplethysmography were revealed in all groups compared to the baseline levels, except for SI in Group 0. A significant increase in IO values was also found after 12 months in Group 0. From the first year of observation, significant differences in the parameters of the functional state of the vessels (PS, IO) were recorded between the groups, while significant differences in the indicators of the structural state (SI, RI), more noticeable in Group 2, became evident only in the third year of observation (Table 6).

Assessments of the capillaroscopy data (Table 7) in Group 0 showed significant dynamics compared to the initial level only for the percentage of perfused capillaries (PPC). Differences in parameters of the functional state of microcirculation (PPC, capillary recovery percentage [CRP]) between the groups were observed from the first year (with the maximum increase in Group 2) and persisted throughout the entire observation period (36 months). For the RCD, no significant intergroup changes were noted after 24–36 months of observation, although they were observed after 12 months.

Applanation tonometry parameters (CASP, rAI), which characterize the structural remodeling of blood vessels, showed significant dynamics within each group compared to their initial values (except for rAI in Group 0; Table 8) but did not demonstrate significant differences between the groups over the study period.

The analysis of the frequency of registered endpoints during the first 12 months of observation did not reveal significant changes for any of them separately (except for CHF-related hospitalizations). The frequency of development of the VCE and OCE was significantly lower in Group 2 than in Groups 1 and 0 (Table 9), while in the placebo counterpulsation group, the VCE and OCE were registered significantly more often than in the EECP groups. After 36 months, the difference between Groups 1 and 2 in terms of the frequency of VCE and OCE events became even more notable.

**Figure 3:**
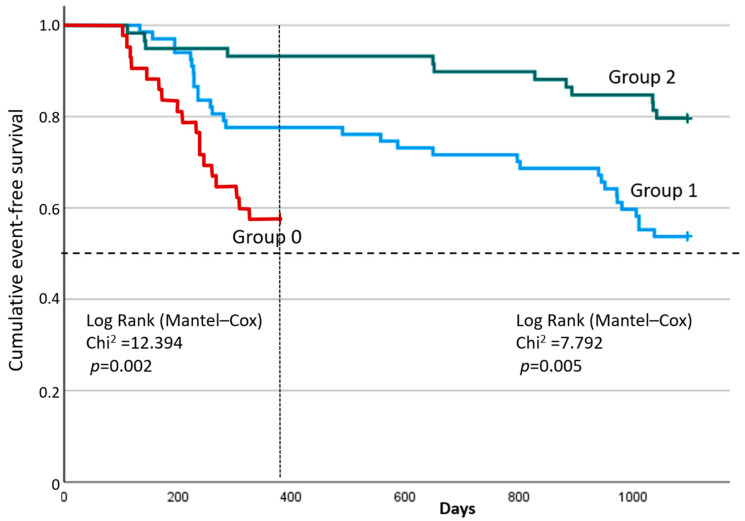
Analysis of event-free survival in the study groups at 12 and 36 months.

A comparative analysis of event-free survival between the study groups using the Log-rank Mantel–Cox test demonstrated statistically significant differences both at 12 months (χ^2^ = 12.394; *p* = 0.002) and at 36 months (χ^2^ = 7.792; *p* = 0.005) (Figure 3). The median survival times in Groups 0, 1, and 2 were not reached at either 12 or 36 months. At 12 months, the 75th percentile of survival time in Group 0 was 229 days from the start of observation (95% CI: 140–∞ days), but the 75th percentiles of survival time in Groups 1 and 2 were not reached. After 36 months, the 75th percentile of survival in Group 1 was 284 days from the beginning of observation (95% CI: 228 –971 days), however, in Group 2 was not reached, which further indicates positive dynamics in this study population. The shape of the curves indicates a better long-term clinical trajectory in Group 2 compared to the others.

**Figure 4:**
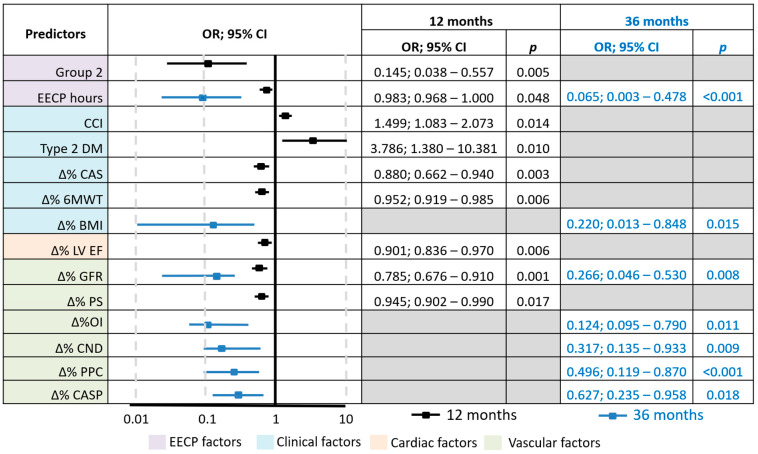
Odds ratio (OR) estimates with 95% confidence intervals (CI) for predictors of achieving the target outcome (OCE) at 12 and 36 months. EECP, enhanced external counterpulsation; CCI, Charlson Comorbidity Index; DM, diabetes mellitus; CAS, Clinical Assessment Scale; 6 MWT, 6 min walk test; BMI, body mass index; LV EF, left ventricular ejection fraction; GFR, glomerular filtration rate; PS, phase shift; OI, occlusion index; CND, capillary network density; PPC, percentage of perfused capillaries; CASP, central aortic systolic pressure.

Binary logistic regression was used to determine the probability of the OCE depending on significant factors (predictors) after 12 and 36 months of observation (Figure 4). The model included 119 observations and was statistically significant (*p* = 0.001) in terms of the correspondence of predicted values to observed values when predictors were included, as compared to the model without predictors. Nigelkirk’s pseudo-R^2^ values were 32.7% and 74.6% for 12 and 36 months, respectively.

**Figure 5:**
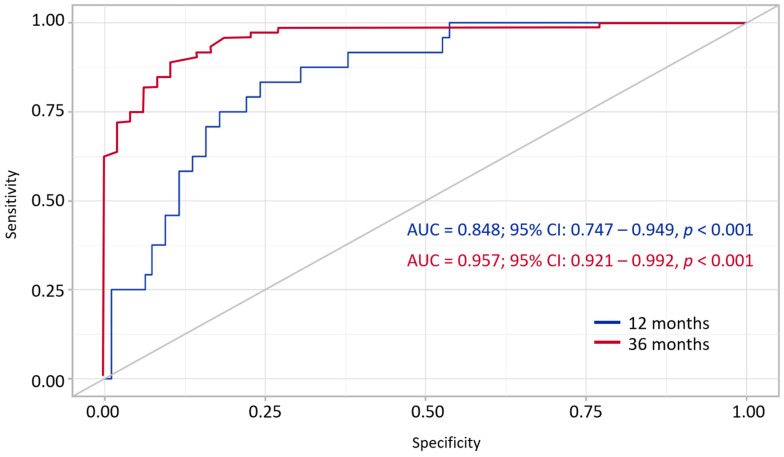
ROC analysis of the model defining the achievement of the target outcome (overall composite endpoint) at 12 and 36 months. AUC, area under curve; CI, confidence interval.

To assess the predictive ability of the developed model in relation to the achievement of the target outcome, an ROC curve characteristic analysis was used (Figure 5).

The definition of probability P is a statistically significant predictor of the OCE after both 12 and 36 months. The area under the ROC curve (AUC) indicates the ability of the model to distinguish between cases where the target condition is achieved. The slope of the ROC curve deviates significantly from the line of equal probability, confirming the diagnostic value of the prognostic model. The optimal threshold probability value was determined based on the maximum value of the Youden index. As a result, the optimal threshold value (for 12 months) was recognized as 0.227, corresponding to a sensitivity of 79.2% and a specificity of 77.9%. At the same time, the positive predictive value (PPV) was 78.2% and the negative predictive value (NPV) was 78.9%, which reflects the balanced quality of the classification. At 36 months, the optimal cutoff value was 0.626, with sensitivity of 88.7% and a specificity of 89.6%.

## 4. Discussion

All patients with ischemic CHF in this study had various disorders of the large vessels (e.g., increased stiffness, endothelial dysfunction, increased CASP) and microcirculatory bed (e.g., capillary density reduction, capillary architecture impairment, increased peripheral resistance, capillary endothelial dysfunction).

The long-term EECP treatment in patients with ischemic CHF, compared to the placebo counterpulsation group, was accompanied by a significantly greater increase in exercise tolerance, an improvement in LV systolic function (increase in LV EF), a reduction in myocardial stress (decrease in NT-proBNP levels), and improvements in both functional (PS, IO, CRP and PPC) and structural (SI, RI, RCD, rAI and CASP) vascular parameters. Moreover, the effect size depended on the number of EECP courses per year (annual number of hours). Notably, functional vascular improvements were observed within the first year, while structural changes emerged during the second or the third year.

In the EECP groups, compared with the placebo counterpulsation group, hospitalizations for CHF and composite endpoints (vascular and overall) were significantly less frequent after 12 months. At the same time, the lowest frequency of these events throughout the study was observed in the group with two EECP courses per year, in which the event-free survival was the highest.

Key predictors of failure to achieve the overall CE after 12 months of observation were the dynamics of clinical factors (Charlson Comorbidity Index, presence of type 2 diabetes, clinical status, exercise tolerance), cardiac (LVEF) and vascular (glomerular filtration rate, PS) factors, and factors associated with EECP treatment (annual number of EECP hours). At 36 months, vascular factors (glomerular filtration rate, IO, CASP, capillary network density, and percentage of perfused capillaries) emerged as predominant predictors, with a decrease in the role of clinical and cardiac factors.

Caceres et al. demonstrated that improved microvascular perfusion during EECP treatment in patients with angina resulted in a significant reduction in angina episodes and improved the patients’ exercise capacity [18]. Moreover, EECP was associated with beneficial effects in patients with diabetic coronary microcirculatory dysfunction [19].

According to S. Lin et al., patients with CHF show significant improvements in cardiac output and stroke volume after EECP, indicating improved cardiac function [20]. In a study by K.M. Tescon et al., 6.1% of patients were rehospitalized within 90 days after EECP treatment, versus the predicted 34.0% [21]. Soran et al. revealed that in patients with refractory angina and LV dysfunction (LV EF < 30 ± 8%), a course of EECP treatment resulted in reductions in emergency department visits (by 78%) and hospitalizations (by 73%) [22]. Comparing the results with earlier studies may be difficult, as those studies were conducted at the end of the last century, when the treatment of patients with CAD and CHF was significantly different (revascularization and medical treatment). Another classification of heart failure was also used. In the study by Soran et al. [22], in the registry included patients recruited in 1998–1999. A total of 1090 (77.7%) of patients had preserved LV function (LVEF > 35%) and 312 (22.3%) had LV dysfunction (LVEF ≤ 35%). Our study included patients with HFrEF (<40%) or HFmrEF (40–49%). At 6 months follow-up, patients with LV dysfunction showed 9.3% deaths, 9.9% exacerbation of congestive heart failure and 15.4% composite outcomes including death/myocardial infarction/coronary artery bypass grafting/percutaneous coronary intervention. In our study, at 12 months follow-up, patients treated with EECP showed no deaths, 2.5% exacerbation of congestive heart failure and 2.5–12.0% vascular composite outcomes. At 36 months follow-up, patients who were treated with EECP showed the rate of 0–3.4% deaths, 11.9–18.6% exacerbation of congestive heart failure, and 6.8–22.0% vascular composite outcomes. Patients in the Soran et al. study [22] underwent fewer revascularizations procedures and received less optimal treatment with medications (compared to our study) and had more severe heart failure. However, Soran et al., like most other authors, studied only a single course of EECP for 6 months or more. According to our data, the vascular effects of EECP persist for 3–6 months, although clinical and cardiac effects may persist for up to 12–18 months.

Microcirculation plays a significant role in maintaining adequate tissue perfusion, which is critical for tissue metabolic processes, especially in patients with CHF [3]. Structural and functional vascular abnormalities are closely interrelated and contribute significantly to the pathophysiology of CHF. Thus, capillary density reduction and endothelial dysfunction can limit the coronary blood flow reserve, which is associated with worse clinical outcomes. Microvascular dysfunction is common in CAD, where it is not limited to the coronary bed but extends to all vessels [23].

The vascular function of patients with CHF is significantly affected by concomitant diseases, systemic inflammation, and insulin resistance [24]. The combination of impaired glucose metabolism and microvascular ischemia exacerbates oxidative stress and mitochondrial dysfunction in the myocardium, requiring additional mechanisms for their correction to improve the results of CHF treatment [25].

In CAD patients, the development of CHF leads to the greater impairment of tissue perfusion due to a decrease in LV systolic function and the further activation of tissue angiotensin systems. The inability of the microcirculation to meet metabolic demands often leads to exercise intolerance and poor functional capacity in patients with CHF, and also contributes to shortness of breath and fatigue, thereby reducing quality of life. Remawi B.N. et al. indicated the need for complex approaches to resolve the problem of microcirculatory dysfunction in patients with CHF and, simultaneously, reduce the overall burden of heart failure [26].

EECP is an additional treatment method for patients with CHF, complementing pharmacological and invasive approaches. The principle of action of EECP is similar to that of an “external heart.” This technique of enhancing blood circulation using external inflatable cuffs placed on the lower extremities is a subject of interest due to its potential to improve tissue perfusion, including myocardial perfusion.

Diastolic retrograde blood flow causes an increase in diastolic perfusion pressure and increasing endothelial shear stress. This effect promotes vasodilation [27] and collateral blood flow [28] by stimulating nitric oxide synthesis, thereby increasing microvascular perfusion not only in ischemic tissue but in all tissues. EECP improves myocardial perfusion, myocardial oxygen delivery and, as a result, exercise tolerance in patients with ischemic CHF [29].

EECP stimulates physiological adaptive mechanisms such as revascularization (due to the development of collaterals), an improvement in endothelial function, a reduction in afterload, an increase in diastolic filling of the LV, and reduction in the defect of mechano-energetic coupling of the myocardium [30,31], which is a benefit of this method.

It is plausible that a long-term improvement in tissue perfusion using EECP in patients with CHF contributes to the enhanced metabolic activity and deactivation of angiotensin systems in tissues, including the myocardium. Therefore, it can be hypothesized that EECP treatment may potentially reduce adverse cardiovascular outcomes and increase the quality and duration of life of these patients.

Certain intervals between EECP courses and performing them regularly may possibly achieve stable vascular and other peripheral effects, as well as cardiac effects.

Most studies on EECP in patients with ischemic CHF have evaluated the effects of only a single course of EECP. Among other limitations, such as small sample sizes, a lack of randomization, and short follow-up periods. These factors significantly reduce the strength of evidence for the effectiveness of EECP, but conducting larger and/or longer studies currently presents methodological challenges.

The limitations of our study include its single-center nature, relatively small sample size, and lack of use of “gold standards” in assessments of functional parameters. In addition, over a long follow-up period, there were difficulties in evaluating the optimal pharmacotherapy, which changed with the advent of subsequent clinical guidelines.

The absence of a double-blind design, changes in pharmacotherapy, which are inevitable in long-term patient observation, the non-central determination of outcomes, and the absence of an independent committee to adjudicate endpoints may affect the validity of the results. The small numbers of events in group comparisons may not be representative enough, which should also be taken into account when interpreting the results.

Despite these limitations, current research data suggest that EECP is a safe, effective, and beneficial treatment option for patients with ischemic CHF and refractory angina. However, further research is needed to optimize EECP treatment protocols [32] and expand the indications for its application.

## 5. Conclusions

Our study has found improvement in vascular, cardiac, and clinical parameters, as well as event-free survival in patients with ischemic CHF in all groups. This improvement was significantly greater in the EECP groups, in addition to drug therapy, compared to placebo-counterpulsation (drug therapy alone). Also, an increase in the observation period allowed us to find an increase in the role of vascular effects in preventing adverse clinical events (combined clinical endpoints). Therefore, EECP should be considered as part of a comprehensive treatment strategy for patients with refractory angina and CHF not suitable for surgical intervention, especially those who have not responded adequately to drug therapy. The inclusion of EECP in standard treatment protocols with CHF patients may provide additional options to relieve symptoms, improve function, reduce the risk of adverse events, and prolong survival in patients with ischemic CHF.

## Figures and Tables

**Table 1:** Indicators of the structural and functional state of the cardiovascular system.

Heart		Large Vessels		Small Vessels	
Function Indicator	Structure Indicator	Function Indicator	Structure Indicators	Function Indicators	Structure Indicators
LV EF ^1^	LV MMI ^1^	PS ^2^	- SI ^2^ - CASP ^4^ - rAI ^4^	- IO ^2^ - PPC ^3^ - CRP ^3^	- RI ^2^ - RCD ^3^

LV EF, left ventricular ejection fraction; LV MMI, left ventricular myocardial mass index; PS, phase shift; SI, stiffness index; CASP, central aortic systolic pressure; rAI, radial augmentation index; IO, occlusion index; PPC, percentage of perfused capillaries; CRP, capillary recovery percentage; RI, reflection index; RCD, resting capillary density. ^1^ echocardiography; ^2^ finger photoplethysmography; ^3^ nailfold capillaroscopy; ^4^ applanation tonometry.

**Table 2:** Basic clinical characteristics in all patient groups (n = 120).

Characteristics	Group 0 (n = 40)	Group 1 (n = 40)	Group 2 (n = 40)	*p*-Value
Age, years	64.1 (57.5; 69.8)	63.5 (56.8; 70.0)	64.0 (57.5; 70.3)	0.534
Men, n (%)	34 (83.6)	31 (77.8)	32 (80.0)	0.347
Duration of CAD (anamnestic), years	7.1 (5.5; 11.2)	7.0 (6.0; 11.0)	6.6 (5.2; 10.7)	0.274
Duration of CHF (anamnestic), years	3.9 (2.5; 7.5)	4.1 (2.2; 6.9)	4.0 (2.6; 7.3)	0.450
HFrEF, n (%)	22 (55.0)	19 (47.5)	21 (52.5)	0.304
HFmrEF, n (%)	18 (45.0)	21 (52.5)	19 (47.5)	
NYHA class II, n (%)	23 (57.5)	26 (65.0)	23 (57.5)	0.732
NYHA class III, n (%)	17 (42.5)	14 (35.0)	17 (42.5)	
Smoking, n (%)	8 (20.0)	6 (15.0)	6 (15.0)	0.190
Multivessel disease, n (%)	18 (45.0)	17 (42.5)	19 (47.5)	0.227
History of MI, n (%)	33 (82.5)	31 (77.5)	30 (75.0)	0.211
History of PCI, n (%)	33 (82.5)	32 (80.0)	32 (80.0)	0.690
History of CABG, n (%)	16 (40.0)	17 (42.5)	16 (40.0)	0.300
Hypertension, n (%)	27 (67.5)	29 (72.5)	27 (67.5)	0.443
Obesity, n (%)	17 (42.5)	17 (42.5)	11 (27.5)	0.636
Type 2 diabetes mellitus, n (%)	20 (50.0)	21 (52.5)	20 (50.0)	0.509
Atrial fibrillation, n (%)	4 (10.0)	4 (10.0)	5 (12.5)	0.398
CKD III–V stage, n (%)	15 (37.5)	12 (30.0)	14 (35.0)	0.267
Charlson Comorbidity Index	5.0 (4.0; 5.0)	5.0 (3.0; 6.0)	5.0 (4.0 6.0)	0.827

CAD, coronary artery disease; CHF, chronic heart failure; HFrEF, heart failure with reduced ejection fraction; HFmrEF, heart failure with mildly reduced ejection fraction; NYHA, New York Heart Association; MI, myocardial infarction; PCI, percutaneous coronary intervention; CABG, coronary artery bypass grafting; CKD, chronic kidney disease.

**Table 3:** Dynamics of exercise tolerance in all patient groups.

6 MWT (m)	**Group 0**	**Group 1**	**Group 2**	
	**Me (Q_1_–Q_3_)**	**Me (Q_1_–Q_3_)**	**Me (Q_1_–Q_3_)**	***p***
Baseline	290 (249–318)	294 (244–324)	292 (232–327)	0.971 ^a^
12 months	310 (275–343)	364 (304–390)	420 (335–463)	<0.001 ^a^
24 months	-	376 (312–415)	458 (387–501)	<0.001 ^b^
36 months	-	381 (324–418)	470 (401–510)	<0.001 ^b^
*p* _12 months_	<0.001 ^c^	<0.001 ^c^	<0.001 ^c^	–
*p* _36 months_	-	<0.001 ^d^	<0.001 ^d^	

^a^ Kruskal–Wallis test; ^b^ Mann–Whitney U test; ^c^ Wilcoxon test; ^d^ Friedman test. 6 WMT, 6 min walk test. Background color indicates statistically significant changes.

**Table 4:** Dynamics of laboratory parameters in all patient groups.

NT-proBNP (pg/mL)	**Group 0**	**Group 1**	**Group 2**	
	**Me (Q_1_–Q_3_)**	**Me (Q_1_–Q_3_)**	**Me (Q_1_–Q_3_)**	***p***
Baseline	931.0 (799.0–1120.8)	999.3 (882.8–1145.5)	1019.4 (906.4–1293.8)	0.062 ^a^
12 months	225.5 (186.4–269.6)	196.2 (174.5–249.1)	180.5 (155.3–200.9)	<0.001 ^a^
24 months	-	155.9 (138.3–198.4)	141.7 (123.3–170.0)	0.003 ^b^
36 months	-	147.8 (131.1–189.1)	138.1 (122.5–164.9)	0.016 ^b^
*p* _12 months_	<0.001 ^c^	<0.001 ^c^	<0.001 ^c^	–
*p* _36 months_	-	<0.001 ^d^	<0.001 ^d^	
GFR (mL/min/1.73 m^2^)	**Me (Q_1_–Q_3_)**	**Me (Q_1_–Q_3_)**	**Me (Q_1_–Q_3_)**	***p***
Baseline	64.5 (53.9–75.2)	66.1 (58.2–77.2)	69.5 (56.6–82.4)	0.103 ^a^
12 months	62.6 (52.9–69.0)	66.2 (58.3–77.4)	75.1 (61.1–89.0)	<0.001 ^a^
24 months	-	65.7 (55.8–75.0)	72.7 (59.3–83.7)	0.030 ^b^
36 months	-	65.7 (56.2–74.8)	70.5 (57.5–82.6)	0.029 ^b^
*p* _12 months_	<0.001 ^c^	0.261 ^c^	<0.001 ^c^	
*p* _36 months_	-	<0.001 ^d^	<0.001 ^d^	

^a^ Kruskal–Wallis test; ^b^ Mann–Whitney U test; ^c^ Wilcoxon test; ^d^ Friedman test; GFR, glomerular filtration rate. NT-proBNP, N-terminal prohormone of brain natriuretic peptide. Background color indicates statistically significant changes.

**Table 5:** Dynamics of echocardiographic parameters in all patient groups.

LV EF (%)	**Group 0**	**Group 1**	**Group 2**	***p***
	**Me (Q_1_–Q_3_)**	**Me (Q_1_–Q_3_)**	**Me (Q_1_–Q_3_)**	
Baseline	34.0 (26.8–45.0)	36.5 (29.0–43.0)	35.0 (29.5–43.0)	0.747 ^a^
12 months	36.0 (7.8–47.3)	40.5 (32.0–48.0)	43.0 (36.5–53.0)	0.018 ^a^
24 months	-	42.1 (32.5–50.5)	48.0 (40.5–54.0)	0.012 ^b^
36 months	-	43.2 (33.5–52.0)	51.0 (43.0–54.0)	0.017 ^b^
*p* _12 months_	<0.001 ^c^	<0.001 ^c^	<0.001 ^c^	–
*p* _36 months_	-	<0.001 ^d^	<0.001 ^d^	
LV MMI (g/m^2^)	**Me (Q_1_–Q_3_)**	**Me (Q_1_–Q_3_)**	**Me (Q_1_–Q_3_)**	
Baseline	113.6 (108.3–119.7)	115.3 (107.4–121.6)	115.7 (107.6–123.5)	0.665 ^a^
12 months	114.3 (108.5–119.7)	114.7 (106.8–121.0)	114.5 (106.6–122.2)	0.580 ^a^
24 months	-	113.9 (107.0–120.1)	113.3 (105.7–119.4)	0.322 ^b^
36 months	-	113.8 (106.9–120.0)	111.1 (103.1–117.0)	0.051 ^b^
*p* _12 months_	<0.001 ^c^	<0.001 ^c^	<0.001 ^c^	
*p* _36 months_	-	<0.001 ^d^	<0.001 ^d^	

^a^ Kruskal–Wallis test; ^b^ Mann–Whitney U test; ^c^ Wilcoxon test; ^d^ Friedman test. LV EF, left ventricular ejection fraction; LV MMI, left ventricular myocardial mass index. Background color indicates statistically significant changes.

**Table 6:** Long-term dynamics of finger photoplethysmography parameters in all patient groups.

	**Group 0**	**Group 1**	**Group 2**	***p***
SI, m/s	**Me (Q_1_–Q_3_)**	**Me (Q_1_–Q_3_)**	**Me (Q_1_–Q_3_)**	
Baseline	8.10 (7.30–8.98)	8.25 (7.42–8.95)	8.45 (7.57–9.60)	0.666 ^a^
12 months	8.10 (7.30–9.00)	8.50 (7.62–9.25)	7.80 (7.00–8.90)	0.417 ^a^
24 months	-	8.00 (7.00–8.70)	7.70 (7.05–8.65)	0.084 ^b^
36 months	-	7.90 (7.00–8.60)	7.00 (6.45–8.00)	0.004 ^b^
*p* _12 months_	0.091 ^c^	<0.001 ^c^	<0.001 ^c^	
*p* _36 months_	-	<0.001 ^d^	<0.001 ^d^	
RI, %	**Me (Q_1_–Q_3_)**	**Me (Q_1_–Q_3_)**	**Me (Q_1_–Q_3_)**	***p***
Baseline	36.8 (33.8–42.3)	35.7 (31.1–42.6)	38.5 (32.4–45.8)	0.495 ^a^
12 months	36.6 (33.6–42.1)	35.0 (30.5–41.8)	37.4 (31.4–44.4)	0.491 ^a^
24 months	-	35.2 (31.3–41.3)	34.4 (29.9–41.1)	0.629 ^b^
36 months	-	34.9 (31.0–40.8)	32.9 (28.2–38.1)	0.048 ^b^
*p* _12 months_	<0.001 ^c^	<0.001 ^c^	<0.001 ^c^	
*p* _36 months_	-	<0.001 ^d^	<0.001 ^d^	
PS, m/s	**Me (Q_1_–Q_3_)**	**Me (Q_1_–Q_3_)**	**Me (Q_1_–Q_3_)**	***p***
Baseline	5.80 (5.33–6.38)	6.15 (5.60–6.50)	5.60 (4.60–6.70)	0.380 ^a^
12 months	5.80 (5.30–6.40)	6.35 (5.80–6.70)	7.30 (5.97–8.72)	<0.001 ^a^
24 months	-	6.15 (5.43–6.60)	7.20 (6.35–8.80)	<0.001 ^b^
36 months	-	6.20 (5.53–6.60)	7.70 (6.80–9.50)	<0.001 ^b^
*p* _12 months_	<0.001 ^c^	<0.001 ^c^	<0.001 ^c^	
*p* _36 months_	-	<0.001 ^d^	<0.001 ^d^	
OI	**Me (Q_1_–Q_3_)**	**Me (Q_1_–Q_3_)**	**Me (Q_1_–Q_3_)**	***p***
Baseline	1.52 (1.35–1.75)	1.56 (1.34–1.64)	1.54 (1.17–1.69)	0.680 ^a^
12 months	1.50 (1.34–1.72)	1.56 (1.35–1.66)	1.69 (1.29–1.85)	<0.001 ^a^
24 months	-	1.58 (1.36–1.72)	1.77 (1.50–1.96)	0.004 ^b^
36 months	-	1.61 (1.39–1.73)	1.80 (1.54–2.00)	0.003 ^b^
*p* _12 months_	<0.001 ^c^	<0.001 ^c^	<0.001 ^c^	
*p* _36 months_	-	<0.001 ^d^	<0.001 ^d^	

^a^ Kruskal–Wallis test; ^b^ Mann–Whitney U test; ^c^ Wilcoxon test; ^d^ Friedman test. SI, stiffness index; RI, reflection index; PS, phase shift; OI, occlusion index. Background color indicates statistically significant changes.

**Table 7:** Long-term dynamics of nailfold videocapillaroscopy parameters in all patient groups.

	**Group 0**	**Group 1**	**Group 2**	***p***
RCD, n/mm^2^	**Me (Q_1_–Q_3_)**	**Me (Q_1_–Q_3_)**	**Me (Q_1_–Q_3_)**	
Baseline	46.0 (43.0–49.0)	46.0 (43.0–48.0)	47.0 (44.5–48.0)	0.951 ^a^
12 months	46.0 (43.0–50.0)	47.0 (44.0–49.0)	52.0 (49.5–53.0)	<0.001 ^a^
24 months	-	46.0 (40.3–50.8)	46.0 (40.5–50.9)	0.866 ^b^
36 months	-	46.0 (40.3–50.8)	46.6 (40.5–51.1)	0.550 ^b^
*p* _12 months_	0.067 ^c^	<0.001 ^c^	<0.001 ^c^	
*p* _36 months_	-	<0.001 ^d^	<0.001 ^d^	
PPC, %	**Me (Q_1_–Q_3_)**	**Me (Q_1_–Q_3_)**	**Me (Q_1_–Q_3_)**	***p***
Baseline	86.8 (83.8–90.2)	85.8 (84.1–89.8)	89.0 (85.2–91.8)	0.118 ^a^
12 months	86.9 (83.9–90.4)	86.7 (85.0–90.7)	93.0 (89.0–95.9)	<0.001 ^a^
24 months	-	87.9 (85.7–92.6)	92.0 (88.7–95.8)	<0.001 ^b^
36 months	-	88.1 (85.8–93.3)	92.5 (89.1–96.2)	<0.001 ^b^
*p* _12 months_	<0.001 ^c^	<0.001 ^c^	<0.001 ^c^	
*p* _36 months_	-	<0.001 ^d^	<0.001 ^d^	
CRP, %	**Me (Q_1_–Q_3_)**	**Me (Q_1_–Q_3_)**	**Me (Q_1_–Q_3_)**	***p***
Baseline	8.80 (6.10–10.60)	8.90 (6.25–11.43)	8.00 (5.97–10.12)	0.690 ^a^
12 months	8.80 (6.10–10.85)	11.05 (7.83–13.30)	14.70 (10.95–18.60)	<0.001 ^a^
24 months	-	10.95 (7.70–14.30)	14.10 (11.25–17.35)	<0.001 ^b^
36 months	-	11.10 (7.83–14.47)	14.20 (11.45–17.50)	<0.001 ^b^
*p* _12 months_	0.052 ^c^	<0.001 ^c^	<0.001 ^c^	
*p* _36 months_	-	<0.001 ^d^	<0.001 ^d^	

^a^ Kruskal–Wallis test; ^b^ Mann–Whitney U test; ^c^ Wilcoxon test; ^d^ Friedman test. RCD, resting capillary density; PPC, percentage of perfused capillaries; CRP, capillary recovery percentage. Background color indicates statistically significant changes.

**Table 8:** Long-term dynamics of applanation tonometry parameters in all patient groups.

	**Group 0**	**Group 1**	**Group 2**	***p***
rAI, %	**Me (Q_1_–Q_3_)**	**Me (Q_1_–Q_3_)**	**Me (Q_1_–Q_3_)**	
Baseline	97.0 (83.0–109.3)	94.6 (86.5–107.6)	94.6 (87.4–103.7)	0.989 ^a^
12 months	97.0 (83.0–109.0)	92.7 (84.8–105.4)	89.9 (83.0–98.5)	0.496 ^a^
24 months	-	92.3 (83.6–104.5)	90.5 (80.2–103.8)	0.455 ^b^
36 months	-	91.8 (82.5–104.0)	85.2 (76.9–98.8)	<0.001 ^b^
*p* _12 months_	0.090 ^c^	<0.001 ^c^	<0.001 ^c^	–
*p* _36 months_	-	<0.001 ^d^	<0.001 ^d^	
CASP, mm Hg	**Me (Q_1_–Q_3_)**	**Me (Q_1_–Q_3_)**	**Me (Q_1_–Q_3_)**	***p***
Baseline	128.5 (120.0–137.8)	126.0 (120.8–130.0)	128.0 (122.0–135.5)	0.280 ^a^
12 months	130.0 (120.0–138.0)	125.0 (119.7–129.0)	125.0 (119.2–132.5)	0.303 ^a^
24 months	-	123.5 (118.3–128.0)	124.0 (119.0–133.0)	0.313 ^b^
36 months	-	123.0 (117.3–127.8)	122.0 (117.0–131.0)	0.181 ^b^
*p* _12 months_	<0.001 ^c^	<0.001 ^c^	<0.001 ^c^	–
*p* _36 months_	-	<0.001 ^d^	<0.001 ^d^	

^a^ Kruskal–Wallis test; ^b^ Mann–Whitney U test; ^c^ Wilcoxon test; ^d^ Friedman test. CASP, central aortic systolic pressure; rAI, radial augmentation index. Background color indicates statistically significant changes.

**Table 9:** Frequency of secondary endpoints in all patient groups.

Parameters	12 months				36 months		
	Group 0 (n = 40)	Group 1 (n = 40)	Group 2 (n = 40)	*p*	Group 1 (n = 59)	Group 2 (n = 59)	*p*
MI, n (%)	2 (5.5)	2 (5.0)	1 (2.5)	0.812	4 (6.8)	0	0.364
PCI/CABG, n (%)	3 (7.5)	3 (7.5)	0	0.232	7 (11.9)	3 (5.2)	0.432
Death, n (%)	1 (2.5)	0	0	0.365	2 (3.4)	0	0.496
Hospitalization due to CHF, n (%)	7 (17.5)	2 (2.5)	1 (2.5)	0.034	11 (18.6)	7 (11.9)	0.443
New-onset AF, n (%)	2 (5.0)	2 (5.0)	0	0.355	4 (6.8)	0	0.119
New-onset type 2 DM, n (%)	2 (2.5)	0	0	0.131	2 (3.4)	0	0.496
New cases of CKD stage 3–5, n (%)	1 (2.5)	1 (2.5)	1 (2.5)	1.000	0	0	1.000
VCE, n (%)	6 (15.0)	5 (12.5)	1 (2.5)	0.039	13 (22.0)	4 (6.8)	0.034
OCE, n (%)	18 (45.0)	10 (25.0)	3 (7.5)	<0.001	31 (52.5)	12 (20.3)	<0.001

MI, myocardial infarction; AF, atrial fibrillation; CHF, chronic heart failure; PCI, percutaneous coronary intervention; CABG, coronary artery bypass grafting; DM, diabetes mellitus; CKD, chronic kidney disease; VCE, vascular composite endpoint; OCE, overall composite endpoint. Background color indicates statistically significant changes.

## Data Availability

The original contributions presented in this study are included in the article.

## Abbreviations

The following abbreviations are used in this manuscript:

EECP	enhanced external counterpulsation
CHF	chronic heart failure
CAD	coronary artery disease
LV	left ventricle
EXCEL	Long-term Effects of enhanced eXternal CountErpuLsation
EF	ejection fraction
OMT	optimal medical therapy
HFrEF	heart failure with reduced ejection fraction
HFmrEF	heart failure with mildly reduced ejection fraction
MMI	myocardial mass index
PS	phase shift
SI	stiffness index
CASP	central aortic systolic pressure
rAI	radial augmentation index
IO	occlusion index
PPC	percentage of perfused capillaries
CRP	capillary recovery percentage
RI	reflection index
RCD	resting capillary density
VCE	vascular composite endpoint
OCE	overall composite endpoint
CABG	coronary artery bypass grafting
CKD	chronic kidney disease
NYHA	New York Heart Association
6 MWT	6 min walk test
GFR	glomerular filtration rate
AF	atrial fibrillation
DM	diabetes mellitus
PCI	percutaneous coronary intervention
CI	confidence interval
OR	odds ratio
AUC	area under the curve
CCI	Charlson Comorbidity Index

## References

[1] Savarese G., Becher P.M., Lund L.H., Seferovic P., Rosano G.M.C., Coats A.J.S. (2023). Global burden of heart failure: A comprehensive and updated review of epidemiology. Cardiovasc. Res..

[2] Russian Society of Cardiology (RSC) (2020). 2020 Clinical practice guidelines for Chronic heart failure. Russ. J. Cardiol..

[3] McMurray J.J., Adamopoulos S., Anker S.D., Auricchio A., Böhm M., Dickstein K., Falk V., Filippatos G., Fonseca C., Gomez-Sanchez M.A. (2012). ESC Guidelines for the diagnosis and treatment of acute and chronic heart failure 2012: The Task Force for the Diagnosis and Treatment of Acute and Chronic Heart Failure 2012 of the European Society of Cardiology. Developed in collaboration with the Heart Failure Association (HFA) of the ESC. Eur. Heart J..

[4] McDonagh T.A., Metra M., Adamo M., Gardner R.S., Baumbach A., Böhm M., Burri H., Butler J., Čelutkienė J., Chioncel O. (2021). 2021 ESC Guidelines for the diagnosis and treatment of acute and chronic heart failure. Eur. Heart J..

[5] Li S., Hao X., Xiao S., Xun L. (2020). The Effect of YiQiFuMai on Ischemic Heart Failure by Improve Myocardial Microcirculation and Increase eNOS and VEGF Expression. Int. J. Clin. Med..

[6] Ahmad A., Corban M.T., Toya T., Verbrugge F.H., Sara J.D., Lerman L.O., Borlaug B.A., Lerman A. (2021). Coronary microvascular dysfunction is associated with exertional haemodynamic abnormalities in patients with heart failure with preserved ejection fraction. Eur. J. Heart Fail..

[7] Kitzman D.W., Nicklas B., Kraus W.E., Lyles M.F., Eggebeen J., Morgan T.M., Haykowsky M. (2014). Skeletal muscle abnormalities and exercise intolerance in older patients with heart failure and preserved ejection fraction. Am. J. Physiol. Heart Circ. Physiol..

[8] Raza A., Steinberg K., Tartaglia J., Frishman W.H., Gupta T. (2017). Enhanced External Counterpulsation Therapy: Past, Present, and Future. Cardiol. Rev..

[9] Wu E., Desta L., Broström A., Mårtensson J. (2020). Effectiveness of Enhanced External Counterpulsation Treatment on Symptom Burden, Medication Profile, Physical Capacity, Cardiac Anxiety, and Health-Related Quality of Life in Patients with Refractory Angina Pectoris. J. Cardiovasc. Nurs..

[10] Jan R., Khan A., Zahid S., Sami A., Owais S.M., Khan F., Asjad S.J., Jan M.H., Awan Z.A. (2020). The Effect of Enhanced External Counterpulsation (EECP) on Quality of life in Patient with Coronary Artery Disease not Amenable to PCI or CABG. Cureus.

[11] Belenkov Y.N., Lishuta A.S., Slepova O.A., Nikolaeva N.S., Khabarova N.V., Dadashova G.M., Privalova E.V. (2024). The EXCEL Study: Long-Term Observation of the Effectiveness of Drug and Non-drug Rehabilitation in Patients with Ischemic Heart Failure. Kardiologiia.

[12] Mitchell C., Rahko P.S., Blauwet L.A., Canaday B., Finstuen J.A., Foster M.C., Horton K., Ogunyankin K.O., Palma R.A., Velazquez E.J. (2019). Guidelines for Performing a Comprehensive Transthoracic Echocardiographic Examination in Adults: Recommendations from the American Society of Echocardiography. J. Am. Soc. Echocardiogr..

[13] Wu M.T., Liu I.F., Tzeng Y.H., Wang L. (2022). Modified photoplethysmography signal processing and analysis procedure for obtaining reliable stiffness index reflecting arteriosclerosis severity. Physiol. Meas..

[14] Belenkov I.N., Privalova E.V., Danilogorskaia I.A., Shchendrygina A.A. (2012). Structural and functional changes in capillary microcirculation in patients with cardiovascular diseases (arterial hypertension, coronary heart disease, chronic heart failure) observed during computer videocapillaroscopy. Russ. J. Cardiol. Cardiovasc. Surg..

[15] Borg A.L., Trapani J. (2024). The accuracy of radial artery applanation tonometry and intra-arterial blood pressure monitoring in critically ill patients: An evidence-based review. Nurs. Crit. Care.

[16] Bubnova M.G., Persiyanova-Dubrova A.L. (2020). Six-minute walk test in cardiac rehabilitation. Cardiovasc. Ther. Prev..

[17] Charlson M.E., Pompei P., Ales K.L., McKenzie C.R. (1987). A new method of classifying prognostic comorbidity in longitudinal studies: Development and validation. J. Chron. Dis..

[18] Caceres J., Atal P., Arora R., Yee D. (2021). Enhanced external counterpulsation: A unique treatment for the “No-Option” refractory angina patient. J. Clin. Pharm. Ther..

[19] Liang J., Wei W., Shi J., Huang H., Wu J., Wu G. (2021). Abstract 13341: Enhanced External Counterpulsation Attenuates Coronary Microcirculation Dysfunction in Coronary Artery Disease with Diabetes: Primary Results of EECP-CMD Study. Circulation.

[20] Lin S., Xiao-Ming W., Gui-Fu W. (2020). Expert consensus on the clinical application of enhanced external counterpulsation in elderly people (2019). Aging Med..

[21] Tecson K.M., Silver M.A., Brune S.D., Cauthen C., Kwan M.D., Schussler J.M., Vasudevan A., Watts J.A., McCullough P.A. (2016). Impact of Enhanced External Counterpulsation on Heart Failure Rehospitalization in Patients with Ischemic Cardiomyopathy. Am. J. Cardiol..

[22] Soran O., Kennard E.D., Kfoury A.G., Kelsey S.F. (2006). Two-year clinical outcomes after enhanced external counterpulsation (EECP) therapy in patients with refractory angina pectoris and left ventricular dysfunction (Report from the International EECP Patient Registry). Am. J. Cardiol..

[23] Taqueti V.R., Di Carli M.F. (2018). Coronary Microvascular Disease Pathogenic Mechanisms and Therapeutic Options: JACC State-of-the-Art Review. J. Am. Coll. Cardiol..

[24] Hilty M.P., Merz T.M., Hefti U., Ince C., Maggiorini M., Pichler Hefti J. (2019). Recruitment of non-perfused sublingual capillaries increases microcirculatory oxygen extraction capacity throughout ascent to 7126 m. J. Physiol..

[25] Ramirez A.Y., Doman E.R., Sanchez K., Chilton R.J. (2025). Impact of insulin resistance and microvascular ischemia on myocardial energy metabolism and cardiovascular function: Pathophysiology and therapeutic approaches. Cardiovasc. Endocrinol. Metab..

[26] Remawi B.N., Preston N., Gadoud A. (2025). Development of a complex palliative care intervention for patients with heart failure and their family carers: A theory of change approach. BMC Palliat. Care.

[27] Xu L., Chen X., Cui M., Ren C., Yu H., Gao W., Li D., Zhao W. (2020). The improvement of the shear stress and oscillatory shear index of coronary arteries during Enhanced External Counterpulsation in patients with coronary heart disease. PLoS ONE.

[28] Liu J.Y., Xiong L., Stinear C.M., Leung H., Leung T.W., Wong K.S.L. (2019). External counterpulsation enhances neuroplasticity to promote stroke recovery. J. Neurol. Neurosurg. Psychiatry.

[29] Zhou Z.F., Wang D., Li X.M., Zhang C.L., Wu C.Y., Muppidi V. (2021). Effects of enhanced external counterpulsation on exercise capacity and quality of life in patients with chronic heart failure: A meta-analysis. Medicine.

[30] Maack C. (2023). Mechanoenergetische Defekte bei Herzinsuffizienz. Herz.

[31] Liao S.F., Li Y.J., Cao S., Xue C.D., Tian S., Wu G.F., Chen X.M., Chen D., Qin K.R. (2024). Hemodynamics of ventricular-arterial coupling under enhanced external counterpulsation: An optimized dual-source lumped parameter model. Comput. Methods Programs Biomed..

[32] Lishuta A.S., Slepova O.A., Nikolaeva N.A., Khabarova N.V., Privalova E.V., Belenkov Y.N. (2024). Effectiveness of different treatment regimens of enhanced external counterpulsation in patients with stable coronary artery disease complicated by heart failure. Ration. Pharmacother. Cardiol..

